# Haematuria in Postrenal Transplant Patients

**DOI:** 10.1155/2015/292034

**Published:** 2015-03-30

**Authors:** Ziting Wang, Anantharaman Vathsala, Ho Yee Tiong

**Affiliations:** ^1^Department of Urology, National University Health System, 5 Lower Kent Ridge Road, Singapore 119074; ^2^Division of Nephrology, Department of Medicine, National University Health System, 5 Lower Kent Ridge Road, Singapore 119074; ^3^National University Centre for Organ Transplantation, National University Health System, 5 Lower Kent Ridge Road, Singapore 119074

## Abstract

Haematuria has a prevalence of 12% in the postrenal transplant patient population. It heralds potentially dangerous causes which could threaten graft loss. It is important to consider causes in light of the unique, urological, and immunological standpoints of these patients. We review the literature on common causes of haematuria in postrenal transplant patients and suggest the salient approach to the evaluation of this condition. A major cause of haematuria is urinary tract infections. There should be a higher index of suspicion for mycobacterial, fungal, and viral infection in this group of immunosuppressed patients. Measures recommended in the prevention of urinary tract infections include early removal of foreign bodies as well as prophylactic antibiotics during the early transplant phase. Another common cause of haematuria is that of malignancies, in particular, renal cell carcinomas. When surgically managing cancer in the setting of a renal transplant, one has to be mindful of the limited retropubic space and the need to protect the anastomoses. Other causes include graft rejections, recurrences of primary disease, and calculus formation. It is important to perform a comprehensive evaluation with the aid of an experienced multidisciplinary transplant team.

## 1. Introduction

Renal transplantation has come a long way since Jaboulay attempted to treat 2 patients with end stage renal failure with a porcine and hircine kidney [1] in 1906. Although his attempts ended in failure, advances in surgical techniques, organ preservation, and immunosuppressant regimes have seen improvement in early graft survival and long term graft function, with 1-year graft survival rates ranging from 80% to 95%. With improved graft and patient survivals, multiple complications can be encountered during the posttransplantation surveillance period, of which haematuria is one of the most common. Haematuria, a condition found in 0.7–3% of the general population [2, 3], has a much higher prevalence in the transplant population [4]. It heralds potential dangerous causes which can potentially threaten graft loss. Hence, it is important to consider causes in the light of the unique urological and immunological standpoints of these patients. We review the literature on common causes of haematuria in postrenal transplant patients and suggest the salient approach to the evaluation of this condition.

Preexisting states of postrenal transplant patients contribute to an increased bleeding tendency, including the use of antiplatelet agents for cardiovascular disease and platelet dysfunction. Immunosuppressants, used for both induction and rejection therapies in renal transplant recipients, were also previously implicated in bleeding diathesis in these patients. Studies have also found that successful kidney transplantation only partially reverses the coagulopathy in patients with chronic renal failure [5] and that many renal transplant patients remained anaemic after operation. Anemia itself promotes bleeding diathesis as circulating red blood cells displace platelets towards the vessel wall. This helps maintain their contact with subendothelium at sites of injury. Red blood cells also enhance platelet function by releasing adenosine diphosphate and inactivating prostacyclin [6]. However, before attributing the causes to anaemia or the inherent coagulopathy of renal transplant patients, it is essential to hunt for other reversible causes of the haematuria.

## 2. Causes of Haematuria

### 2.1. Infections

The use of immunosuppressants predisposes patients to urinary tract infections, which can be heralded by the sign of haematuria. In a prospective study performed on patients after kidney transplantation [7], it was found that 37% of patients developed a urinary tract infection, with recurrent infections being observed in 13.4%. With recurrent acute graft pyelonephritis (APGN), it is essential to consider anatomic abnormalities such as strictures at the ureterovesical junction, neurogenic bladder, and vesicoureteral reflux in patients [8], which may necessitate early surgical correction.

With regard to graft prognosis, a discrepancy of opinions exists on the impact of APGN on renal transplant outcome. Some studies found that early APGN is associated with graft loss [9] whilst others suggest that APGN has no impact on graft or recipient survival [10, 11].

Apart from the garden-variety bacterial infections, there should be a higher index of suspicion for mycobacterial, fungal, and viral infection in this group of immunosuppressed patients. Fungal organisms associated with hemorrhagic cystitis include* Candida albicans*,* Cryptococcus*, and* Aspergillus fumigates* [12]. A persistence of candiduria in spite of appropriate antifungals should prompt further investigations in the realm of imaging and target biopsies, looking out for an aspergilloma or abscess. The occurrence of sterile pyuria should also alert one to the possibility of acid fast bacilli infection, of which the polymerase chain reaction is both a sensitive and a specific test to look for both typical and atypical mycobacterium.

The BK virus, adenovirus,* Cytomegalovirus*, and herpes virus have been identified as causation agents of viral hemorrhagic cystitis [13, 14]. These opportunistic organisms remain dormant in the healthy individual after the initial infection. With the use of immunosuppressants, the patient becomes more susceptible to the reactivation of these viruses. Risk factors significantly associated with virus-associated nephropathy include prior transplant rejection, the use of mycophenolate, tacrolimus, antithymocyte globulin agents, and male gender [15]. A reduced immunosuppression regimen is the mainstay of treatment but could prompt rejection. Screening protocol employing the use of plasma nucleic acid testing for early detection of infection and appropriate antiviral agents can help to prevent irreversible kidney damage [16, 17].

However, the old adage remains, “Prevention is better than cure.” Preventative measures recommended in the prevention of urinary tract infections include early removal of foreign bodies including stents and in-dwelling catheters as well as daily trimethoprim-sulfamethoxazole or nitrofurantoin [18] for those with allergies or G6PD deficiencies for at least 6 months after transplantation during the early transplant phase.

### 2.2. Malignancies

Kidney transplant patients are at greater risk of developing certain malignancies, in particular cancers which are associated with viral infections, including human papillomavirus with cutaneous malignancies and EBV with posttransplant lymphoproliferative diseases. The use of immunosuppressive agents which cause DNA damage, impair immune surveillance, and interfere with normal DNA repair has also been implicated in the process of mutagenesis and the development of cancer [19]. Less well-defined in the process of carcinogenesis are the roles of preexisting cancer risk factors and factors related to chronic renal impairment and dialysis.

With special reference to urological cancers, the incidence of renal cell carcinoma is much higher than that in the general population, with a standardized incidence ratio greater than 5. The majority of guidelines do not advocate routine screening as there is a lack of evidence that screening reduces mortality [18, 20]. However, with the new presentation of haematuria, a careful evaluation of the entire urinary tract for malignancies is crucial. Risk factors for the development of renal cell carcinoma include a history of prior renal cell carcinoma, tuberous sclerosis, polycystic kidney disease, and the duration of dialysis before transplant [21, 22].

**Figure 1:**
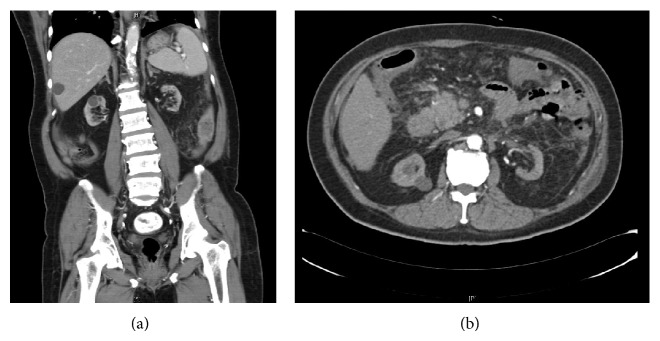
CT findings showing a 1.3 × 1.3 cm enhancing lesion at the upper pole of right kidney.

**Figure 2:**
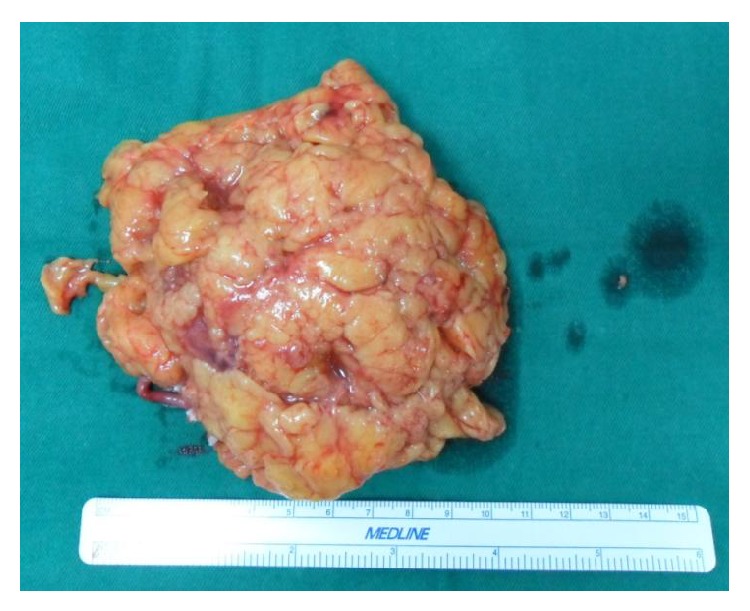
Resection specimen of the papillary renal cell carcinoma.

Figures 1 and 2 show the CT findings and resection specimen of a patient with papillary renal cell carcinoma from malignant change of a cyst in a cystic kidney patient after kidney transplantation.

Malignancy of the native kidney occurs more frequently than the occurrence of cancer in the kidney allograft, with rates of 4.2% and 0.07%, respectively [23, 24]. For renal cell carcinoma in the native kidney, the tumors are usually small and the nephrectomies are amenable to laparoscopic treatment [25, 26]. Regarding the transplant allograft, partial nephrectomy is a dialysis-sparing option for localised tumours less than 4 cm in size [27]. However, the surgery, conducted in a nonvirgin area with fibrous tissue and inflammation, makes mobilisation and resection challenging. Clinical judgement should be made, balancing the considerations of the patient's residual allograft function while not sacrificing surgical margins and oncological control.

There is some evidence that mTOR inhibitors such as Everolimus and its parent drug Sirolimus have antineoplastic activities. In a randomized controlled trial investigating the de novo malignancies arising in patients receiving immunosuppressive agents, the incidence in patients on Sirolimus was significantly lower than the other drugs [28, 29]. This has been attributed to the angiogenesis pathway blockade of the mTOR inhibitors. Previous literature advised against the initiation of mTOR inhibitors in the early postoperative period due to concerns of wound dehiscence, incisional hernia, and lymphocele formation. However, new studies have presented evidence that lower doses of mTOR inhibitors and the avoidance of a loading dose with the concomitant use of other immunosuppressants have significantly reduced the risks of impaired wound healing. Hence, the decision of their use should only be made after a careful consideration of available strategies to prevent surgical complications, individual patient characteristics, and risk factors [30, 31].

Gross haematuria is one of the main presenting symptoms of bladder carcinoma in renal transplant patients [24]. An analysis of the USRDS database using Medicare billing claims for cancer found that the incidence of bladder cancer is three times that of the general population, with the greatest risk occurring in the first 6 years after transplantation [32]. In addition, data suggest that muscle-invasive and high grade tumours are more common amongst renal transplant recipients. One modifiable risk factor is the intake of Aristolochic Acid. A study of 1429 Chinese patients who received renal transplantation found that 59.3% of the patients who were subsequently diagnosed with transitional cell carcinoma had been taking the Chinese herb for at least 2 months prior to diagnosis [33]. Since 2001, the Food and Drug Administration (FDA) has cautioned against the intake of this traditional medication in view of worldwide epidemiological evidence linking Aristolochic Acid exposure and transitional cell carcinoma [34].

For noninvasive disease, transurethral resection of the bladder tumour (TURBT) is the gold standard for first line treatment. The use of BCG and Mitomycin C as adjuvant intravesical chemotherapy is more controversial. Literature demonstrates superior disease recurrence and progression rates for BCG, but the possible induction of a systemic inflammatory response has led some to shy away from the use of this live attenuated strain of* Mycobacterium bovis* in the immunosuppressed group of patients. Others advocate the coadministration of antituberculosis drugs or ciprofloxacin together with intravesical BCG [35, 36].

Management of muscle-invasive bladder carcinoma includes aggressive extirpative surgery and urinary reconstructive options. Ileal conduit urinary diversion is preferable for patients with some graft dysfunction. Orthotopic neobladder is an option for patients with relatively good creatinine clearance and offers continence. We performed a literature search on renal transplant patients who developed bladder carcinoma.  Table 1 documents the patient demographics and treatment regime of the case series.

There is an estimated twofold increase in the risk of prostate carcinoma occurrence in the first 3 years after transplantation [37]. However, data suggests that the incidence subsequently drops to become similar to that of the general population [7]. Hence, the authors advocate following local or regional clinical practice guidelines for prostate cancer screening in the general population.

When managing prostate cancer in the setting of a renal transplant, one has to be mindful of the limited retropubic space and the need to protect the vascular and ureterovesical anastomosis. However, good outcomes have been reported with both open radical prostatectomy and minimally invasive approaches with robotic surgery. There have been a few cases of treatment with radiotherapy and androgen deprivation [38, 39]. Due to the location of the allograft, doses are usually reduced to prevent reported complications of proteinuria, acute and chronic renal failure [40]. Another issue is the distal transplant ureter, which is at risk of developing a stricture, owing to the proximity of the ureterovesical anastomosis to the radiation field. Ensuring that the patient has a full bladder during the time of irradiation provides a more constant position of the distal ureter and reduces its exposure to radiation.

Studies illustrating the outcomes of prostate cancer treatment in renal transplant patient are few owing to the relatively low prevalence of such occurrences. A comparison of oncological outcomes between open radical, robot-assisted radical prostatectomy and radiotherapy is shown in Table 2. Prospective studies and comparison of functional outcomes are lacking.

### 2.3. Rejections

Chronic rejection of the transplanted kidney typically presents with microscopic haematuria, although gross haematuria has been documented in isolated case reports [41]. A study of 1060 renal transplant recipients with haematuria in Korea using evaluation modalities of plain X-ray, sonography, cystoscopy, or graft biopsy found chronic rejection in 18 patients and acute rejection in 5 [42]. While invasive urological investigations are preferably avoided, in the setting of persistent haematuria with no other cause and graft dysfunction, it may be wise to perform early biopsy of the kidney to diagnose rejection and determine severity so that treatment can be initiated.

### 2.4. Disease Recurrences

Haematuria is a common manifestation of glomerulonephritis recurrence, especially with those which present with a primarily nephritic picture, such as Goodpasture's syndrome, systemic lupus erythematosus, and Ig A nephropathy. Data from the Australia and New Zealand Dialysis and Transplant Registry (ANZDATA) showed that recurrence of the primary disease is amongst the top 3 reasons for allograft loss in recipients with glomerulonephritis, with the first and second being chronic rejection and death with a functioning allograft [43], with studies reflecting a recurrence rate of 10–19.4% and a resultant graft loss of up to 50% amongst these patients [44]. The risk of recurrence of primary diseases differs in individual conditions. The prevalence of condition and the disease mechanism have to be taken into account in the workup of haematuria.

Immunoglobulin A nephropathy (IgAN) is one of the most common causes of primary glomerulonephritis worldwide. Most studies underestimate the recurrence of IgAN as most biopsies are only performed in symptomatic patients. Regular screening biopsies performed in transplant patients found a histological recurrence rate of 30–60%, with resultant graft loss reported to be 3–9% [45–48].

Most nephrologists believe that recurrent IgA largely follows a benign course; however, studies have found that allograft survival rate drops after the initial 5 years after transplant [49]. Angiotensin converting enzyme inhibitor and angiotensin receptor blocker can help to reduce proteinuria and preservation of renal function in patients with IgAN [50]. Some studies have found that mycophenolate as an immunosuppressant was associated with a lower risk of IgA nephropathy as opposed to steroid-free and Sirolimus-based regimes [51].

Recurrence rates in focal and segmental glomerulosclerosis (FSGS) have been reported to be as high as 50% [52]. Primary FSGS recurs commonly early in the first 3-4 weeks after transplantation, presenting with heavy proteinuria. However, it should be noted that the urinary protein levels should be compared to the baseline as proteinuria secondary to the primary disease can take a period of time to resolve. The incidence of haematuria in posttransplant FSGS recurrences is not known in literature. However, as 45–55% of patients with primary FSGS have haematuria [53], it is relevant to consider the option of a renal biopsy when such a patient presents with haematuria after transplant. Risk factors for recurrence include a younger age of onset, aggressive disease with development of stage 5 CKD in less than 3 years, mesangial hypercellularity of native kidney, and a history of recurrence leading to a prior graft failure. The last factor is a significant contributor of recurrence risk, with reported rates of up to 80%. Hence, current consensus is that such risks should be conveyed to both recipient and donor and dedicated counselling should be performed accordingly, especially in cases of living donor transplant.

With regard to secondary glomerulonephritis, literature has reported a recurrence rate of 2–10% in lupus nephritis [54, 55]. However, the ANZDATA study did not find any allograft loss due to recurrence of lupus nephritis, Goodpasture's syndrome, and Alport's disease. This suggests that Australian transplantation protocol of postponing transplantation till the disease is unequivocally quiescent could help to lower the prevalence of recurrences in these conditions.

Early diagnosis would enable the physician to initiate interventional therapy to achieve remissions of the glomerulonephritis and help to extend allograft survival. Routine biopsy protocols are still controversial. However, most transplant centres would advocate a monitoring practice consisting of permutation and combinations of creatinine clearance, urine dipstick, microscopy, albumin, or protein creatinine ratio. Any haematuria, significant deterioration in renal function, or proteinuria should precipitate a graft biopsy including immunofluorescence and electron microscopy.

### 2.5. Calculus

The prevalence of calculi in the renal tract ranges from 0.2 to 2% [56, 57]. Most of them are found in the bladder but papers have implicated sutures at the site of the ureteroureterostomy and the ureteroneocystostomy to be possible nidus for calculi formation [56, 58]. Stone composition is largely similar to that of the general population, with calcium stone accounting for the majority of stones formed. Unlike the general population, renal calculi often present with painless haematuria in renal transplant recipients, due to the denervation of the allograft during procurement. Hence, in the presence of strong risk factors such as recurrent urinary tract infections, renal tubular acidosis, and hypercalciuria [59], it is important to perform imaging as an early step in the evaluation of haematuria.

While plain X-rays can diagnose a certain percentage of stones, renal calculi of varying radiolucency can be easily missed, especially since the allograft overlies the iliac bone. A noncontrast computed tomography of the urinary tract is sensitive but costly and subjects the patient to radiation. Many centers support the employment of ultrasonography to identify stones and possible obstruction.

Prompt diagnosis and the initiation of medical or surgical interventions are crucial in averting a compromise in renal graft function. Conservative treatment such as alpha agonists and urine alkalinisation can be employed for small stones less than 4 mm in size, with regular follow-ups looking out for obstruction. For stones that are unable to pass spontaneously or larger stones up to 15 mm, extracorporeal shockwave lithotripsy (ESWL) has been shown to have satisfactory outcomes. Special provisions in the ESWL technique have to be made in view of the position of the pelvic allograft. These include moderating the energy of the shockwaves and number of shocks employed, placing the patient in a prone position, and employing ureteral stents to aid in stone localization [60, 61].

Compared to the noninvasive ESWL method, ureteroscopic lithotripsy has the advantage of removal of renal and ureteric calculi, although retrograde access can be challenging and ancillary instruments such as the Kumpe catheter may be required to aid in cannulation [62]. In the setting of larger stones, percutaneous antegrade techniques can allow direct access for disintegration of the stone and adequate drainage [63]. A ureteral catheter is first maneuvered to be just proximal to the ureteropelvic junction. The patient is then placed in a supine oblique position with the aid of a bolster under the ipsilateral hemipelvis. The use of the ultrasound probe can both help in visualisation of the target calyx and also displace intervening bowel loops [64].

### 2.6. Others

Haematuria can present from cyst bleeding in a patient with autosomal dominant polycystic kidney disease (ADPKD). Studies have shown that 16–26% of renal transplant patients who have ADPKD subsequently underwent native nephrectomy [65, 66]. There have been many successful cases of a nephrectomy of the native kidneys and renal transplant being performed simultaneously can now be performed to treat complications from the large polycystic kidneys; hence the option of a combined surgery should be offered to patients with recurrent haematuria or intractable pain prior to the transplant. Alternatively, if recurrent haematuria secondary to the polycystic kidneys presents after the transplant, early studies in laparoscopic nephrectomy outcomes have shown promise in reducing blood loss and hospital stay lengths [67, 68].

It is also important to consider the occurrence of haematuria in relation to any recent diagnostic or interventional procedures. Haematuria can occur after percutaneous nephrolithotomies or nephrostomies. There are also case reports documenting the formation of a pseudoaneurysm formation after biopsy [69, 70]. In severe cases, angioembolization may be required to cease the bleeding.

## 3. Conclusion

In view of the high prevalence of haematuria in postrenal transplant patients, the greater likelihood of urological malignancies, and procedure-dependent evaluation, it is pertinent to have a comprehensive evaluation and an experienced multidisciplinary transplant team consisting of the urologist, nephrologist, radiologist, and renal transplant coordinator involved in the follow-up of the patient.

## Figures and Tables

**Table 1:** Patient demographics and treatment regime of renal transplant patients who developed bladder carcinoma.

Comparison	Rogers et al., BJUI, UK, 2012 [71]	Prabharasuth et al., J Urol, USA, 2013 [72]	Moses et al., Transplant Proc, USA, 2013 [73]	Master et al., J Urol, USA, 2004 [24]	Manassero et al., Transplant International, Italy, 2011 [74]	Kamal et al., BJU, Egypt, 2008 [35]	Tomaszewski, AIU, USA, 2011 [36]	Lang et al., J Urol, Germany, 2005 [75]
*N*	8	17	5	5	4	7	7	4

Stage	pTa-T2	pTa-T3	pT0-T3	pT1-T3a	pT1-T3a	pTis-T3a	pTis-T3a	pTa-T3b

Mean time to development of TCC after transplant (months)	60	88.1	83.9	106.8	102	112.8	39	126

Treatment	TURBT (7), RCIUD (1)	RCIUD (4), RCNB (1) TURBT (7), Chemo (3), palliative RT (1)	RCNB (5)	TURBT (1), RCIUD (1), RCNB (2)	RCNB (4)	RCNB (5), TURBT (2)	Palliative RT + IUD (1), palliative RT (1), RCIUD (2), TURBT (3)	RCNB (4)

Mean follow-up (months)	144	9.2	24.9	33.6	31.5	10.3	36.3	52

Recurrence rate	2/8	8/16 (1 palliative RT)	2/5	—	2/4	3/7	2/5 (2 palliative RT)	1/4

TURBT: transurethral resection of bladder tumour; RC: radical cystectomy; IUD: incontinent urinary diversion; NB: neobladder.

**Table 2:** Oncological outcomes of prostate cancer patients.

Comparison	Polcari et al., J Urol, USA, 2009 [76]	Antonopoulos et al., J Urol, Brazil, 2008 [77]	Kleinclauss et al., J Urol, France, 2008 [78]	Mouzin et al., Transplantation, France, 2004 [79]	Elkentaoui et al., J Urol, France, 2010 [80]	Detti et al., JJ Clinical Onco, Italy, 2011 [81]
*N*	7	8	20	8	15	1

Clinical stage	pT1c-T2a	pT2a-T2c	pT2a-T3b	pT1c-T3a	pT2a-T3a	pT3b

Treatment	Robot-assisted radical prostatectomy	Radical prostatectomy	Radical prostatectomy	Three-dimensional conformal radiotherapy	Radical prostatectomy	Radical prostatectomy + adjuvant RT

Mean operative duration (min)	186	183	163	—	—	—

Mean blood loss (mL)	—	656	516	—	—	—

Mean hospital stay (days)	1.8	3	11.9	—	—	—

Postprocedure complications	42.9%	0	0	5/8 grades 1-2 cystitis	2/15 rectal injuries	Grade 1 cystitis

Mean follow-up (months)	16	10.5	23	28	26	—

Recurrence rate	1/7	0	2/20	2/8	1/15	0/1

Positive margins	2/7	2/8	2/20	—	0/15	1/1
